# MicroRNA-382-5p aggravates breast cancer progression by regulating the RERG/Ras/ERK signaling axis

**DOI:** 10.18632/oncotarget.12338

**Published:** 2016-09-29

**Authors:** Jar-Yi Ho, Ren-Jun Hsu, Jui-Ming Liu, Szu-Chi Chen, Guo-Shiou Liao, Hong-Wei Gao, Cheng-Ping Yu

**Affiliations:** ^1^ Department of Pathology, and Graduate Institute of Pathology and Parasitology, Tri-Service General Hospital, National Defense Medical Center, Taipei, Taiwan; ^2^ Biobank Management Center of Tri-Service General Hospital, National Defense Medical Center, Taipei, Taiwan; ^3^ Graduate Institute of Life Sciences, National Defense Medical Center, Taipei, Taiwan; ^4^ Division of Urology, Department of Surgery, Taoyuan General Hospital, Ministry of Health and Welfare, Taoyuan, Taiwan; ^5^ Department of Surgery, Tri-Service General Hospital, National Defense Medical Center, Taipei, Taiwan

**Keywords:** miR-382-5p, RERG, Ras/ERK pathway, breast cancer

## Abstract

Aberrant activation of the Ras/ERK pathway mediates breast cancer initiation and aggressiveness. Therefore, it is important to identify miRNAs that modulate the Ras/ERK pathway during breast carcinogenesis and progression. The Ras GTPase superfamily member RERG (Ras-related and estrogen-regulated growth inhibitor) acts as a tumor suppressor to reduce breast cancer cell proliferation and tumor formation and has been suggested to have a regulatory role in the Ras/ERK pathway. In this study, we found that RERG exerted its tumor suppressor role by attenuating the activation of Ras/ERK signaling effectors. Furthermore, we found that miR-382-5p directly targets and represses RERG to attenuate the inhibitory effects of RERG on the oncogenic Ras/ERK pathway. Thereby, miR-382-5p promoted breast cancer cell viability, clonogenicity, survival, migration, invasion and *in vivo* tumorigenesis/metastasis. In clinical interpretation, miR-382-5p expression was negatively correlated with RERG expression, and it also significantly functioned as an independent oncomiR for the higher incidence and poorer prognosis of breast cancer. This novel connection highlights new diagnostic and prognostic roles for miR-382-5p and RERG in breast cancer.

## INTRODUCTION

Breast cancer is the most frequently diagnosed female cancer and represents the second most common cause of female cancer-related deaths in the world [1]. Furthermore, it accounts for the most morbidity and the fourth highest mortality of female cancers in Taiwan [2]. The Ras/ERK pathway (also known as the mitogen-activated protein kinase (MAPK)/ERK pathway or the Ras-Raf-MEK-ERK cascade) has been reported to play an essential role in tumorigenesis and cancer progression [3, 4]. Hyperactivation of the Ras/ERK pathway has been reported in approximately half of breast cancer cases [5, 6] and significantly associated with the aggressive behavior and poorer prognosis of breast cancer [7–9]. In addition, Ras/ERK pathway hyperactivation is a common feature of a variety of tumor types with activating mutations in *KRAS*, *NRAS*, or *BRAF* [10]; however, mutations in these genes were detected in only ~3.2% of all breast lesions [11]. Nevertheless, most Ras/ERK signaling molecules are often overexpressed in breast cancer [12], suggesting that there are other genomic and/or epigenetic regulations involved in the aberrant activation of the Ras/ERK pathway [5, 7, 13].

MicroRNAs (miRNAs) are small non-coding RNAs with approximately 20-24 nucleotides in the mature form, and they post-transcriptionally suppress other RNAs to regulate diverse cellular processes [14]. In the context of cancer, miRNAs that target oncogenes or tumor suppressor genes are termed tumor suppressor miRs (miRsupps) or oncomiRs, respectively [15, 16]. And miRNA dysregulation has been extensively documented in several carcinogenic breast cancer processes [17]. Several miRNAs have been reported to target members of the RAS/ERK pathway [18], and dysregulation of those miRNAs in cancer cells likely contributes to tumorigenesis by aberrantly activating the RAS/ERK pathway. However, little research has focused on the regulation of miRNAs targeting RAS/ERK signaling modulators in breast cancer. To date, let-7 has been reported to target *HRAS* [19] and *KRAS* [20], miR-200c and miR-30c target *KRAS*, miR-148b targets *NRAS*, miR-7 targets *RAF1*, miR-206 targets *RASA1* and miR-21 targets *SPRED1* in breast cancer cells [18]. Nevertheless, miRNAs that target the upstream regulators of the Ras/ERK pathway have not been studied.

In this study, we performed a miRNA microarray to compare miRNA expression profiles between breast cancer and normal breast specimens, and we found that miR-382-5p had the highest tumor/normal expression ratio among the screened miRNAs. Bioinfomatic analysis predicted that miR-382-5p targeted *RERG* (Ras-related and estrogen-regulated growth inhibitor), a gene encoding a Ras superfamily small GTP binding and hydrolyzing protein (GTPase). RERG has been reported to play a tumor suppressor role in the inhibition of breast cancer cell proliferation and tumor formation [21]. Hence, we examined the regulatory roles of miR-382-5p and RERG in breast carcinogenesis and progression. Our findings suggested that RERG exerted its tumor suppressor role by deactivating Ras/ERK signaling effectors. And miR-382-5p exerted its oncogenic regulation by directly targeting and repressing RERG, thereby activating the Ras/ERK pathway to promote cell viability and more aggressive breast cancer behaviors. Clinically, miR-382-5p significantly functioned as an oncomiR for higher incidence and poorer prognosis of breast cancer. Our findings highlighted the importance of the miR-382-5p/RERG/Ras/ERK axis in driving breast carcinogenesis and progression.

## RESULTS

### MiR-382-5p is upregulated in breast cancer and is associated with breast cancer incidence and progression

**Figure 1:**
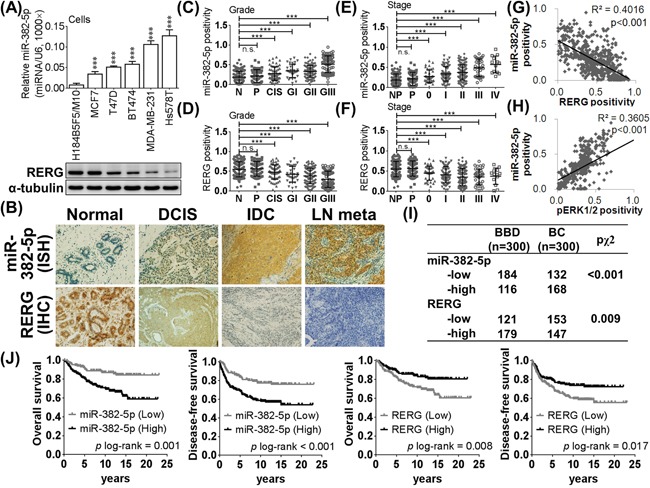
MiR-382-5p is upregulated in breast cancer and was negatively corrected with RERG **A**. The relative expression levels of miR-382-5p were detected by the stem-loop based miRNA real-time PCR (normalized to U6) in breast cell lines (H184B5F5/M10 was used as the baseline of comparison, * *p* < 0.05, ** *p* < 0.01, *** *p* < 0.001, Student *t*-test). And RERG expression levels of each breast cancer cells were evaluated with western blotting. **B**. The expression levels of miR-382-5p and RERG were detected by *in situ* hybridization and immunohistochemistry in formalin-fixed paraffin-embedded breast specimens, respectively. Representative expression profiles of miR-382-5p and RERG were showed among benign breast disease, carcinoma *in situ*, primary breast cancer and lymph node metastatic breast cancer. **C**. MiR-382-5p revealed an increasing trend, but **D**. RERG revealed a decreasing trend among higher pathological grade. **E**. MiR-382-5p revealed an increasing trend, but **F**. RERG revealed a decreasing trend among higher clinical stage cacners. **G**. MiR-382-5p expression profile was negatively correlated with RERG, but **H**. positively correlated with pERK1/2. **I**. The distribution of either miR-382-5p or RERG was siginificantly different between benign breast disease controls and breast cancer cases (Figure 1I, *p* < 0.001 for miR-382-5p, and *p* = 0.009 for RERG, chi-square test). **J**. Kaplan-Meier curves of overall survival (OS) and disease-free survival (DFS) were analyzed with log-rank test. And lower miR-382-5p (*p* = 0.001 for OS and *p* < 0.001 for DFS) and higher RERG (*p* = 0.008 for OS and *p* = 0.017 for DFS) were associated with favorable survival. Abbrev: B (BBD): benign breast disease, BC: breast cancer, CIS: carcinoma *in situ*, DCIS, GI: infiltrating ductal carcinoma grade I, GII: infiltrating ductal carcinoma grade II, GIII: infiltrating ductal carcinoma grade III, LN meta: lymph node metastatic breast cancer.

To identify the crucial miRNAs involved in breast cancer of Taiwanese patients, we screened for the most upregulated oncomiRs in breast cancer using miRNA microarray with stem-loop real-time PCR validation. We found that miR-382-5p was the most upregulated miRNA in the breast cancer specimens (Supplementary Table S1). Similarly, miR-382-5p was significantly more highly expressed in poorer differentiated breast cell lines compared to the normal human breast epithelial cell line H184B5F5/M10 (Figure 1A). To identify the potential target genes of miR-382-5p, we performed bioinformatic predictions using the TargetScanHuman and miRBase databases. There were 27 candidates predicted in both databases, but only RERG was reported as a tumor suppressor gene in breast cancer (Supplementary Figure 1A). Consequently, RERG was expressed in a reciprocal pattern with miR-382-5p, as RERG was expressed at lower levels in more poorly differentiated breast cancer cell lines compared to H184B5F5/M10 cells (Figure 1A).

We performed a case-control association study design in the clinical interpretation, and miR-382-5p was nearly undetectable in benign breast diseases, slightly increased in carcinoma *in situ* and strongly positive in invasive and metastastic cancer cells. In contrast, RERG was significantly downregulated in carcinoma *in situ*, invasive and metastastic breast cancer cells, and more highly expressed in benign breast diseases (Figure 1B). The miR-382-5p expression pattern revealed increasing trends among higher pathological grade (Figure 1C) and higher clinical stage cancers (Figure 1E); however, we observed decreasing trends in the RERG expression pattern (Figure 1D and 1F). MiR-382-5p expression was negatively correlated with RERG expression (Figure 1G), but interestingly, it was also positively correlated with pERK1/2, the predominant active effectors of the Ras/ERK pathway (Figure 1H, Supplementary Figure 1B). In addition, the distribution of either miR-382-5p or RERG was significantly different between benign breast disease controls and breast cancer cases (Figure 1I, *p* < 0.001 for miR-382-5p, and *p* = 0.009 for RERG, chi-square test).

We subsequently evaluated the association between miR-382-5p, RERG and breast carcinogenic risk factors. Univariate analysis revealed significantly higher miR-382-5p and lower RERG in breast cancer patients, and those breast cancer patients had a higher diagnosed age, body mass index and family history. However, there was no difference between cancer cases and controls with respect to other risk factors. After adjustment of miR-382-5p, RERG, diagnosed age, body mass index and family history in multivariant analysis, we identified miR-382-5p as an independent oncomiR of breast cancer carcinogenesis, that is, higher miR-382-5p carriers possessed a 2.063-fold (95% CI 1.044-4.080) higher risk of breast cancer than lower miR-382-5p carriers (Table 1).

**Table 1:** Univariant and multivariant analyses of miR-382-5p, RERG and breast cancer risk factors

Subject characteristics	BC (n=300)	BBD (n=300)	Univariate		Multivariate	
			OR (95%CI)	*p*^a^	OR (95% CI)	*p*^a^
**Diagnostic markers^b^**						
miR-382-5p low	132	184	1 (ref)		1 (ref)	
high	168	116	**2.019 (1.458-2.796)**	**<0.001**	**2.063 (1.044-4.080)**	**0.037**
RERG high	147	179	1 (ref)		1 (ref)	
low	153	121	**1.540 (1.115-2.127)**	**0.009**	1.242 (0.625-2.466)	0.536
**Demographic factors**						
Age ≦51 y/o	117	141	1 (ref.)		1 (ref.)	
>51 y/o	183	159	**1.387 (1.003-1.919)**	**0.048**	1.234 (0.715-2.130)	0.450
Education less than high school	110	103	1 (ref.)			
high school and above	145	161	1.185 (0.794-1.769)	0.407	-	-
Unknown	45	36				
**Reproductive risk factors**						
Age at menarche	13.77±1.69	13.53±1.43	1.110 (0.958-1.285)	0.165	-	-
Age at menopause	48.86±4.93	47.69±6.84	1.036 (0.989-1.084)	0.136	-	-
Total menstruation (y)	35.52±6.29	34.11±6.76	1.035 (0.986-1.085)	0.161	-	-
Age at FFTP	25.68±5.56	26.55±4.85	0.967 (0.921-1.016)	0.183	-	-
No. pregnancy	2.31±1.71	2.64±1.92	1.110 (0.965-1.276)	0.145	-	-
No. live births	2.02±1.27	2.09±1.39	1.160 (0.969-1.389)	0.106	-	-
Lactation <6 month	158	163	1 (ref)			
≧6 month	98	102	0.990 (0.614-1.625)	0.957	-	-
Unknown	44	35				
**Other risk factors**						
Body mass index (BMI, kg/m^2^)	23.06±3.52	22.16±2.58	**1.105 (1.016-1.202)**	**0.019**	1.156 (0.787-1.270)	0.118
Waist-hip ratio (WHR)	0.76±0.59	0.75±0.54	1.092 (0.944-6.538)	0.716	-	-
HRT (−)	211	230	1 (ref)			
(Ever used) (+)	53	59	1.056 (0.399-2.790)	0.913	-	-
Unknown	36	11				
Oral contraceptive (−)	232	247	1 (ref)			
(Ever used) (+)	25	20	0.471 (0.149-1.482)	0.198	-	-
Unknown	43	33				
Cigarette consumption None	240	259	1 (ref)			
>6 months	17	8	2.298 (0.802-6.588)	0.121	-	-
Unknown	43	33				
**Family history**						
None	208	239	1 (ref)		1 (ref.)	
With family history	34	22	**1.776 (1.007-3.132)**	**0.047**	1.122 (0.488-2.583)	0.450
Unknown	58	39				

*^a^* On the basis of logistic regression, the statistical significance (*p*<0.05) is shown in bold.

BBD comprised 237 nonproliferative lesions (124 fibroadenoma, 107 fibrocystic changes, 6 adenosis) and 63 proliferative diseases without atypia (44 mild ductal hyperplasia and 19 sclerosing adenosis). BC comprised 61 ductal carcinoma *in situ*, 27 infiltrating ductal carcinoma (IDC) grade I (GI), 110 IDC GII, 74 IDC GIII, 18 infiltrating lobular carcinomas (ILC), 7 colloid carcinomas, and 3 medullary carcinomas. The miR-382-5p expression profiles were evaluated using *in situ* hybridization and RERG expression profiles were evaluated using immunochemistry.

^b^ Categorization as low (≤mean) or high (>mean) was stratified as miR-382-5p > 28% positivity (high) and miR-382-5p <= 28% positivity (low) and RERG > 46% positivity (high) and RERG <= 46% positivity (low).

Abbrev: BC: breast cancer, BBD: benign breast diseases, FFTP: first full-term pregnancy, HRT: hormone replacement therapy, OR: odds ratio, CI: confidence interval.

To evaluate the prognostic values of miR-382-5p and RERG, we performed survival analyses in breast cancer patients. Higher miR-382-5p or lower RERG was significantly associated with poorer overall survival (OS) and disease-free survival (DFS) of breast cancer (Figure 1J; *p* < 0.05). Cox proportional hazard regression models suggested that higher miR-382-5p, lower RERG, and higher clinical stages or higher pathological grades were also significantly associated with poorer OS and DFS (Table 2). After adjustment for miR-382-5p, RERG, clinical stages and pathological grades by multivariant analysis, we identified higher clinical stage and pathological grades as independent risk factors for the poorer prognosis of breast cancer. Similarly, higher miR-382-5p also acted as an independent oncomiR for the poorer prognosis of breast cancer. Higher miR-382-5p carriers showed a 2.148-fold higher hazard ratio for breast cancer-associated death and 2.226-fold higher hazard ratio for breast cancer progression compared to lower miR-382-5p carriers (Table 2). Moreover, the association between higher miR-382-5p and poorer prognosis was similar in both ERα (+) and ERα (−) breast cancer subtypes (Supplementary Table S2 and S3). Taken together, miR-382-5p expression is a predictive and prognostic oncomiR for the higher incidence and poorer prognosis of breast cancer.

**Table 2:** Survival assay of miR-382-5p, RERG and clinical prognostic factors of breast cancer

	n	Overall survival				Disease-free survival			
		Univariate^a^		Multivariate^a^		Univariate^a^		Multivariate^a^	
		HR (95% CI)*^a^*	*p*	HR (95% CI)*^a^*	*p*	HR (95% CI)*^a^*	*p*	HR (95% CI)*^a^*	*p*
miR-382-5p low	132	1 (ref)		1 (ref)		1 (ref)		1 (ref)	
high	168	**2.688 (1.562-4.556)**	**0.001**	**2.148 (1.069-4.318)**	**0.032**	**2.213 (1.433-3.416)**	**0.001**	**2.226 (1.238-4.001)**	**0.007**
RERG high	147	1 (ref)		1 (ref)		1 (ref)		1 (ref)	
low	153	**1.915 (1.176-3.116)**	**0.009**	1.003 (0.530-1.897)	0.629	**1.633 (1.087-2.452)**	**0.018**	1.321 (0.758-2.302)	0.326
Age ≦53 y/o	126	1 (ref)				1 (ref)			
>53 y/o	174	1.449 (0.908-2.312)	0.120	-	-	1.043 (0.697-1.560)	0.840	-	-
Family history (−)	208	1 (ref)				1 (ref)			
(+)	34	1.251 (0.494-3.171)	0.637	-	-	1.054 (0.476-2.332)	0.897	-	-
unknown	58	**-**	**-**	**-**	**-**	**-**	**-**	**-**	**-**
TNM stage 0+I+II	248	1 (ref)		1 (ref)		1 (ref)		1 (ref)	
III+IV	52	**9.776 (6.045-15.777)**	**<0.001**	**7.828 (4.757-12.880)**	**<0.001**	**7.030 (4.658-10.610)**	**<0.001**	**5.639 (3.701-8.593)**	**<0.001**
Histologic grade CIS+G1	92	1 (ref)		1 (ref)		1 (ref)		1 (ref)	
G2+G3	208	**3.117 (1.596-6.089)**	**0.001**	1.763(0.875-3.552)	0.112	**3.171 (1.800-5.587)**	**0.001**	**2.155 (1.199-3.873)**	**0.010**
ER (+)	207	1 (ref)				1 (ref)			
(−)	93	0.968 (0.585-1.601)	0.899	-	-	0.978 (0.636-1.502)	0.918	-	-
PR (+)	205	1 (ref)				1 (ref)			
(−)	95	1.106 (0.664-1.841)	0.698	-	-	1.012 (0.661-1.549)	0.957	-	-
HER2 (+)	96	1 (ref)				1 (ref)			
(−)	204	1.094 (0.669-1.789)	0.721	-	-	1.342 (0.892-2.019)	0.159	-	-
Chemotherapy (−)	137	1 (ref)				1 (ref)			
(+)	163	0.995 (0.622-1.591)	0.983	-	-	1.143 (0.764-1.710)	0.516	-	-
Radiotherapy (−)	171	1 (ref)				1 (ref)			
(+)	129	0.839 (0.522-1.347)	0.467	-	-	0.840 (0.565-1.249)	0.389	-	-
Hormone therapy (−)	121	1 (ref)				1 (ref)			
(+)	179	1.037 (0.642-1.675)	0.882	-	-	1.104 (0.732-1.665)	0.636	-	-

^a^ Based on Cox regression model, statistical significance (*p*< 0.05) is shown in bold.

TNM, tumor node metastasis; HR, hazard ratio; CI, confidence interval.

^b^ Categorization was also stratified as low (≤mean) and high (>mean). Abbrev: HR: hazard ratio, CI: confidence interval.

### MiR-382-5p promotes breast cancer cell viability, clonogenicity, survival, migration and invasion

**Figure 2:**
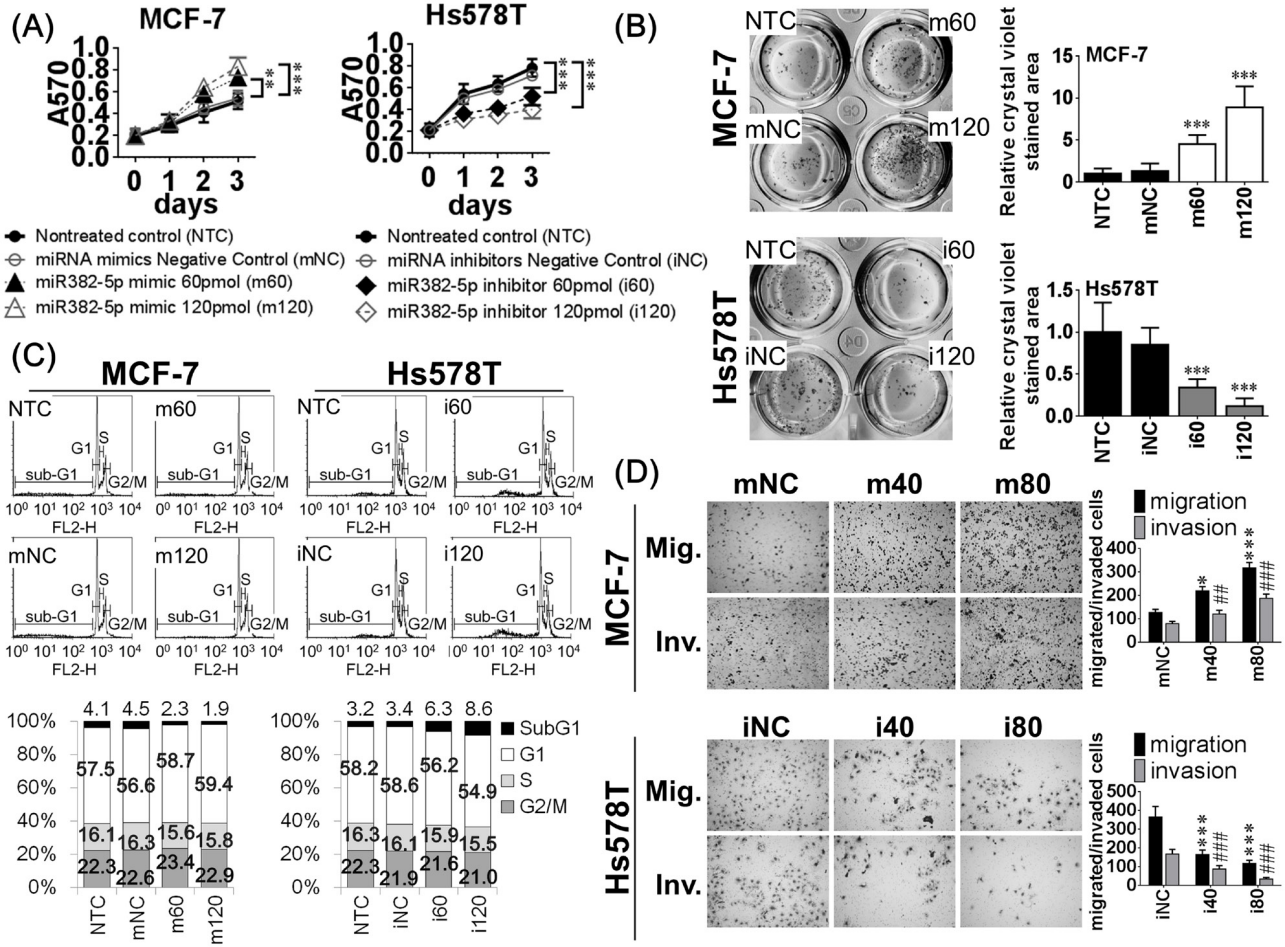
The biological effects of miR-382-5p were evaluated in MCF-7 cells (lower endogenous miR-382-5p) and Hs578T cells (higher endogenous miR-382-5p) **A**. MCF-7 cells were treated with four different conditions, non-treated control (NTC), 120 pmol miRNA mimics negative control (mNC), 60 pmol miR-382-5p mimics (m60), and 120 pmol miR-382-5p mimics (m120). And Hs578T cells were treated with other four conditions, non-treated control (NTC), miRNA inhibitors negative control (iNC), 60 pmol miR-382-5p inhibitors (i60), and 120 pmol miR-382-5p inhibitors (i120). After transfection, cells were maintained for 72 h and the cell viability was determined using MTT assays. MCF-7 cells transfected with miR-382-5p mimics revealed dose-dependent induction of cell viability than NTC or mNC groups. Conversely, Hs578T cells transfected with miR-382-5p inhibitors revealed a dose-dependent reduction of cell viability than NTC or iNC groups (Student *t*-tests were analyzed at 72 h, * *p* < 0.05, ** *p* < 0.01, *** *p* < 0.001). **B**. Cell clonogenicity was evaluated with colony-formation assays, and miR-382-5p mimics dose-dependently induced colony-forming ability than NTC or mNC groups. Conversely, miR-382-5p inhibitors dose-dependently reduced colony-forming ability than NTC or iNC groups. The bar-charts were used to show the relative crystal violet stained area which were normalized to the NTC group (* *p* < 0.05, ** *p* < 0.01, *** *p* < 0.001, Student *t*-test). **C**. MiR-382-5p effect on apoptosis was evaluated with PI-stained flow cytometry. And MCF-7 cells transfected with miR-382-5p mimics revealed a dose-dependent reduction of sub-G1 population compared to NTC or mNC groups. Conversely, Hs578T cells transfected with miR-382-5p inhibitors revealed a dose-dependent induciton of sub-G1 population compared to NTC or iNC groups. The bar-charts were used to show the relative percentage of each phase of cell cycle. **D**. MiR-382-5p effects on cell migratory and invasive abilities were analyzed with transwell and Matrigel-coated transwell assays, respectively. We used lower concentrations of miR-382-5p mimics or inhibitors to reduce the interference of apoptosis. MCF-7 cells transfected with miR-382-5p mimics revealed a dose-dependent increase of both migrated and invaded cells compared to NTC or mNC groups. Conversely, Hs578T cells transfected with miR-382-5p inhibitors revealed a dose-dependent reduction of both migrated and invaded cells compared to NTC or iNC groups. The bar-charts were used to show the migrated/invaded cell numbers which were normalized to the NTC group (* *p* < 0.05, ** *p* < 0.01, *** *p* < 0.001 for cell migration and # *p* < 0.05, ## *p* < 0.01, ### *p* < 0.001 for cell invasion, Student *t*-test).

To evaluate the biological effects of miR-382-5p, we performed gain-of-function and loss-of-function studies in MCF-7 cells (lower endogenous miR-382-5p) and Hs578T cells (higher endogenous miR-382-5p, Figure 1A). MTT assays and soft-agar colony-formation assays were used to compare the effects of miR-382-5p on cell viability and clonogenicity. MCF-7 cells transfected with miR-382-5p mimics revealed a dose-dependent induction of cell viability than non-treated controls (NTC) or miRNA mimic negative controls (mNC) groups (Figure 2A). Similarly, miR-382-5p dose-dependently induced a higher colony-forming ability than NTC or mNC groups (Figure 2B). Conversely, Hs578T cells transfected with miR-382-5p inhibitors attenuated endogenous miR-382-5p and dose-dependently decreased cell viability (Figure 2A) and colony-forming ability (Figure 2B) than NTC or miRNA inhibitor negative control (iNC) groups. These data suggest that miR-382-5p enhanced cell viability and may induce higher tumorigenicity, at least in an experimental cell model.

We evaluated the effect of miR-382-5p on apoptosis by performing flow cytometric analysis of PI-stained cells. MCF-7 cells transfected with miR-382-5p mimics revealed a dose-dependent reduction of the sub-G1 population compared to NTC or mNC groups. Conversely, Hs578T cells transfected with miR-382-5p inhibitors revealed a dose-dependent induction of the sub-G1 population compared to the NTC and iNC groups (Figure 2C). To compare the effects of miR-382-5p on cell motility, we performed transwell and Matrigel-coated transwell assays to evaluate cell migration and invasion, respectively. We used lower concentrations of miR-382-5p mimics or inhibitors to reduce the interference of apoptosis. MCF-7 cells transfected with miR-382-5p mimics revealed a dose-dependent increase in migration and invasion compared to NTC and mNC groups. Conversely, Hs578T cells transfected with miR-382-5p inhibitors revealed a dose-dependent reduction in migration and invasion compared to NTC and iNC groups (Figure 2D). Therefore, miR-382-5p also reduced cancer cell apoptosis and promoted cell migration and invasion.

### MiR-382-5p directly targets the RERG 3′UTR

**Figure 3:**
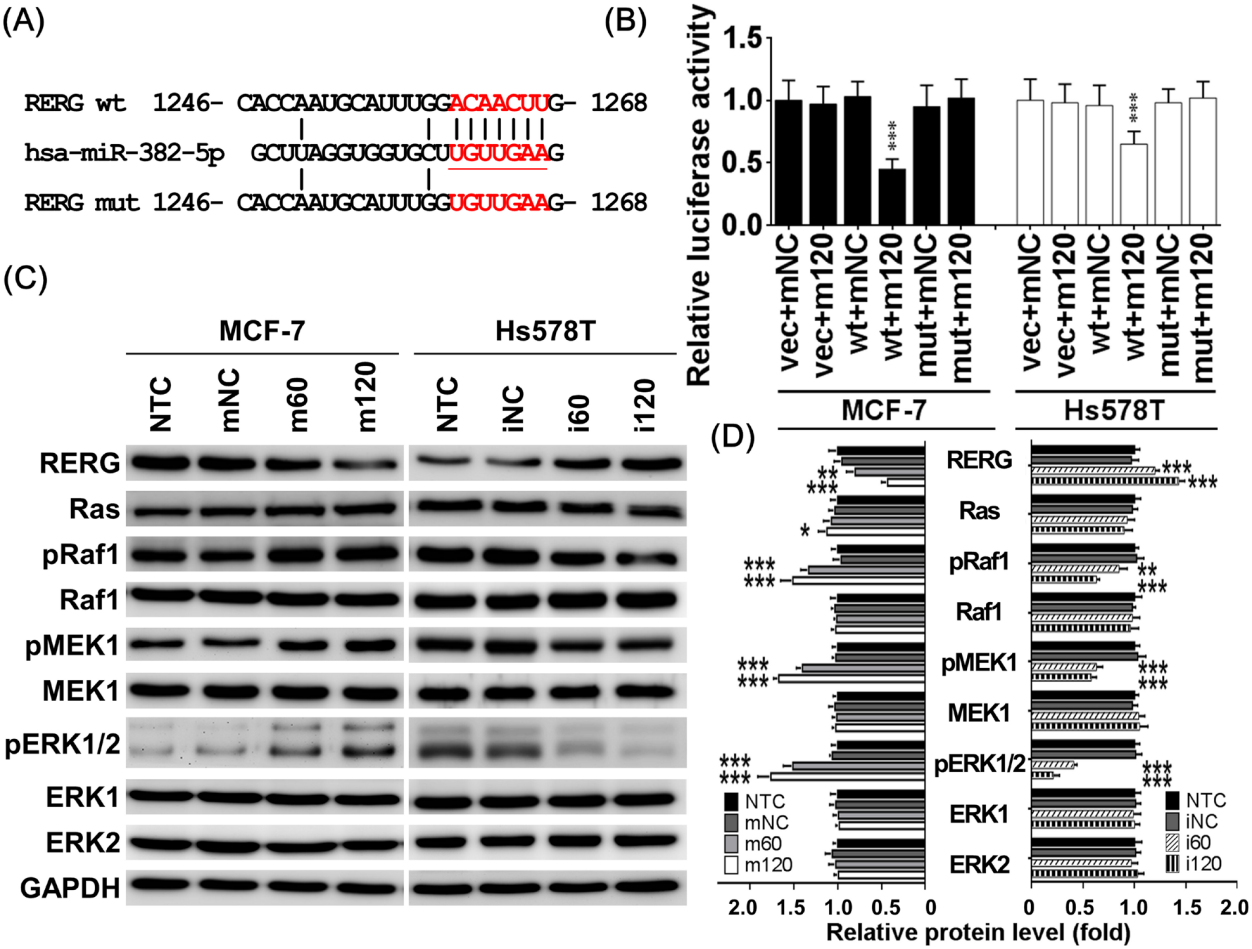
MiR-382-5p directly targeted 3′UTR of RERG **A**. The predicted miR-382-5p targeting site within the RERG 3′UTR and the mutated sites were aligned as indicated. **B**. After co-transfection with the miR-382-5p mimics or mNC, the relative luciferase activity of pMIR-Reporter carrying the wild-type or mutant RERG 3′UTRs was determined in MCF-7 and Hs578T cells and normalized to the empty vector transfectants. β-Galactosidase control vector was also co-transfected and used as an transfection loading control. MiR-382-5p mimics reduced luciferase activity in cells transfected with p-MIR-Reporter carrying the wild-type RERG 3′UTR compared to mNC-transfected cells. There was no change in luciferase activity in cells co-transfected with miR-382-5p mimics or mNC with empty vector alone or p-MIR-Reporter carrying amutant RERG3′UTR. **C**. The protein levels of Ras/ERK signaling members were determined using western blotting in MCF-7 and Hs578T cells treated with the same conditions of Figure 2A-2C. And miR-382-5p mimics dose-dependently reduced RERG expression and activated Ras/ERK signaling effectors, pRaf1, pMEK1/2, and pERK1/2 in MCF-7 cells, but miR-382-5p inhibitors dose-dependently resuced RERG expression and deactivated pRaf1, pMEK1/2, and pERK1/2 in Hs578T cells. **D**. The bar-charts were used to show the relative protein levels which were normalized to GAPDH and the NTC group was used as the comparative baseline (* *p* < 0.05, ** *p* < 0.01, *** *p* < 0.001, Student *t*-test).

The predicted miR-382-5p targeting site within the RERG 3′UTR is shown in Figure 3A. We performed luciferase reporter assays to validate RERG as a direct miR-382-5p target gene. We found that miR-382-5p mimics reduced luciferase activity in cells transfected with p-MIR-Reporter carrying the wild-type RERG 3′UTR compared to mNC-transfected cells. However, there was no change in luciferase activity in cells co-transfected with miR-382-5p mimics or mNC with empty vector alone or p-MIR-Reporter carrying amutant RERG3′UTR (Figure 3B). In addition, miR-382-5p mimics had a less repressive effect on Hs578T cells in which endogenous miR-382-5p is already higher compared to other breast cancer cell lines (Figure 1A).

To confirm the specificity of the miR-382-5p suppressive effect on RERG, we performed western blotting to compare the expression of RERG and major Ras/ERK pathway members. As expected, MCF-7 cells transfected with miR-382-5p mimics expressed significantly lower levels of RERG protein. Consequently, miR-382-5p mimics slightly increased Ras protein levels, but remarkably increased its downstream effectors, including pRaf1, pMEK1/2, and pERK1/2. These data suggest that RERG downregulation resulted in higher activation of the Ras/ERK pathways (Figure 3C, 3D). Conversely, Hs578T cells transfected with miR-382-5p inhibitors expressed significantly higher levels of RERG protein and lower levels of the Ras/ERK signaling effectors pRaf1, pMEK1/2, and pERK1/2 (Figure 3C, 3D). RERG is a Ras-related GTPase, and its active form has been suggested to have a tumor suppressor role. We found that RERG not only reduced Ras activation but also slightly reduced its stability, though not to statistically significant levels. Taken together, these results implicated that miR-382-5p suppresses RERG by directly targeting its 3′UTR, and thereby reduces Ras/ERK pathway activation.

### Effects of RERG knockdown and overexpression

**Figure 4:**
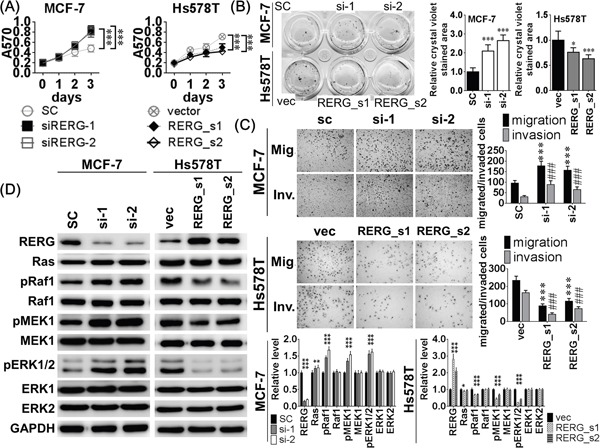
Knockdown of RERG resulted in similar inhibitory effects of miR-382-5p but RERG overexpression reduced the inductive effects of miR-382-5p **A**. MCF-7 cells were treated with 120 pmol scrambled miR control (SC), 120 pmol siRERG-1 (si-1), and 120 pmol siRERG-2 (si-2) for 72 h, and the cell viability was determined using MTT assays. And RERG siRNAs increased cell viabilitycompared to the SC group. Conversely, pcDNA3-RERG was stably transfected into Hs578T and two stable clones (RERG_s1 and RERG_s2) were selected to compare cell viability with empty vector transfected control (vec). Overexpression of RERG reduced cell viability compared to the vec group (*** *p* < 0.001, Student *t*-test). After treated with the same conditions for 72 h, cells were conducted to **B**. soft-agar colony-formation assays and **C**. transwell or Matrigel-coated transwell assays. And RERG knockdown also promoted cell clonogenicity, migration and invasion compared to the SC group (B: *p < 0.05, *** *p* < 0.001; and C: *** *p* < 0.001 for migration, ### *p* <0.001 for invasion, Student *t*-test). **D**. The protein levels of the Ras/ERK signaling members were determined using western blotting. RERG knockdown reduced RERG expression but activated Ras/ERK signaling effectors, pRaf1, pMEK1/2, and pERK1/2 in MCF-7 cells, Overexpression of RERG increased RERG expression but deactivated pRaf1, pMEK1/2, and pERK1/2 in Hs578T cells. The bar-charts were used to show the relative protein levels which were normalized to GAPDH and the SC and vec groups were used as the comparative baselines in MCF-7 and Hs578T cells, respectively (* *p* < 0.05, *** *p* < 0.001, Student *t*-test).

To confirm the inhibitory effects of RERG on the Ras/ERK pathway, we transfected RERG siRNA into MCF-7 cells and pcDNA3-RERG into Hs578T cells to evaluate the biological effects of either RERG knockdown or overexpression. RERG siRNA increased cell viability and colony-forming ability compared to the scrambled control (SC) group (Figure 4A, 4B), and they also reduced the sub-G1 cell population compared to the SC group (Supplementary Figure 1C). In addition, MCF-7 cells transfected with RERG siRNAs had higher migratory and invasive abilities in transwell assays than the SC groups (Figure 4C). RERG siRNAs also increased the amount of phosphorylated Ras/ERK signaling effectors, pRaf1, pMEK1/2, and pERK1/2, demonstrating the activation of the pathway (Figure 4D). Conversely, RERG overexpression in Hs578T cells reduced cell viability and colony-forming ability in RERG stably transfected clones compared to vector (vec) control groups (Figure 4A, 4B). RERG stably transfected Hs578T cells also had an increased sub-G1 cell population compared to the SC group (Supplementary Figure 1C). Moreover, RERG stably transfected Hs578T cells were less migratory and invasive (Figure 4C), and expressed lower levels of pRaf1, pMEK1/2, and pERK1/2 than the vec groups (Figure 4D). These results suggest that RERG knockdown led to similar suppressive effects of miR-382-5p to increase Ras/ERK signaling activation and related biological effects, but RERG overexpression inhibited Ras/ERK signaling activation and related biological effects.

### MiR-382-5p enhanced cell viability and aggressive behaviors, which are attenuated by RERG expression

**Figure 5:**
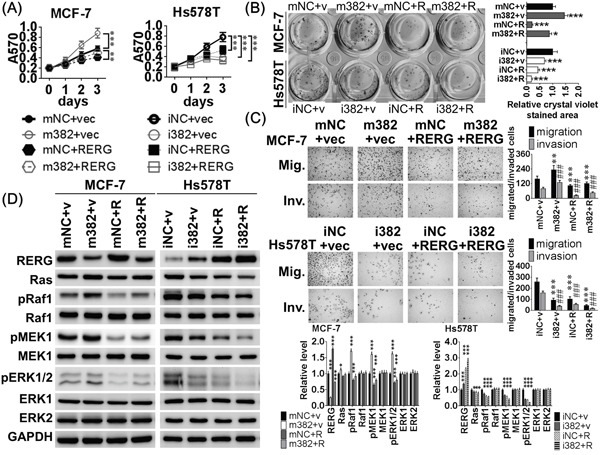
MiR-382-5p enhanced cell viability and aggressive behaviors, which are attenuated by RERG expression **A**. MCF-7 cells were transiently co-transfected with four combinations for 72 h: 120 pmol miRNA mimics negative control and 2 μg pcDNA3.1 empty vector (mNC+vec), 120 pmol miR-382-5p mimics and 2 μg pcDNA3.1 empty vector (m382+vec), 120 pmol miRNA mimics negative control and 2 μg pcDNA3-RERG (mNC+RERG), and 120 pmol miR-382-5p mimics and 2 μg pcDNA3-RERG (m382+RERG). The inductive effects of miR-382-5p on (A) cell viability, **B**. clonogenicity, **C**. migration and invasion were impaired in MCF-7 cells co-transfected with m382+RERG. The growth curves and bar-charts were used to show the relative levels which were normalized to the mNC+vec groups (A&B: * *p* < 0.05, ** *p* < 0.01, *** *p* < 0.001; and C: ** *p* < 0.01, *** *p* < 0.001 for migration, ### *p* <0.001 for invasion, Student *t*-test). Hs578T cells were also transiently transfected with other four combinations for 72h: 120 pmol miRNA inhibitors negative control and 2 μg pcDNA3.1 empty vector (iNC+vec), 120 pmol miR-382-5p inhibitors and 2 μg pcDNA3.1 empty vector (i382+vec), 120 pmol miRNA inhibitors negative control and 2 μg pcDNA3-RERG (iNC+RERG), and 120 pmol miR-382-5p inhibitors and 2 μg pcDNA3-RERG (i382+RERG). Hs578T co-transfected with i382+RERG additively reduced (A) cell viability, (B) clonogenicity, (C) migration and invasion. The growth curves and bar-charts were used to show the relative levels which were normalized to the iNC+vec groups (A&B: *** *p* < 0.001, and C: *** *p* < 0.001 for migration, ### *p* <0.001 for invasion, Student *t*-test). **D**. The protein levels of the Ras/ERK signaling members were determined using western blotting. And MCF-7 cells co-transfected with m382+RERG attenuated miR-382-5p induced Ras/ERK signaling effectors, pMEK1/2 and pERK1/2. Otherwise, Hs578T cells co-transfected with i382+RERG rescued RERG expression and additively deactivated pRaf1, pMEK1/2, and pERK1/2. The bar-charts were used to show the relative protein levels which were normalized to GAPDH and the mNC+vec or iNC+vec groups were used as the comparative baseline in MCF-7 and Hs578T cells, respectively (* *p* < 0.05, ** *p* < 0.01, *** *p* < 0.001, Student *t*-test).

To examine whether RERG compensative expression attenuated the oncogenic effects of miR-382-5p on breast cancer cell viability and progression, we co-transfected pcDNA3-RERG or empty vector with miR-382-5p mimics or mNC into MCF-7 cells. Western blot analysis confirmed that miR-382-5p mimics markedly and specifically decreased RERG expression, but pcDNA3-RERG-transfected MCF-7 cells overexpressed RERG protein (Figure 5D). The inductive effects of miR-382-5p on cell viability, clonogenicity, migration and invasion were impaired in MCF-7 cells co-transfected with miR-382-5p mimics and pcDNA3-RERG (Figure 5A–5C). Co-transfection of miR-382-5p mimics and pcDNA3-RERG also significantly attenuated the miR-382-5p-induced activation of Ras/ERK signaling effectors (Figure 5D).

We also performed an additive experiment in which we co-transfected pcDNA-RERG (versus the vec group) and the miR-382-5p inhibitor (versus the iNC group) into Hs578T cells, and found that RERG protein levels were rescued after transfection with the miR-382-5p inhibitor (Figure 5D). The repressive effects of miR-382-5p inhibitors on cell viability, clonogenicity, migration and invasion increased in Hs578T cells co-transfected with miR-382-5p inhibitors and pcDNA3-RERG (Figure 5A–5C), and co-transfection significantly reduced Ras/ERK signaling effector activation (Figure 5D). Taken together, these findings revealed that RERG is a direct and functional target of miR-382-5p in Ras/ERK signaling regulation.

### MiR-382-5p promotes *in vivo* breast cancer tumorigenesis and metastasis

**Figure 6:**
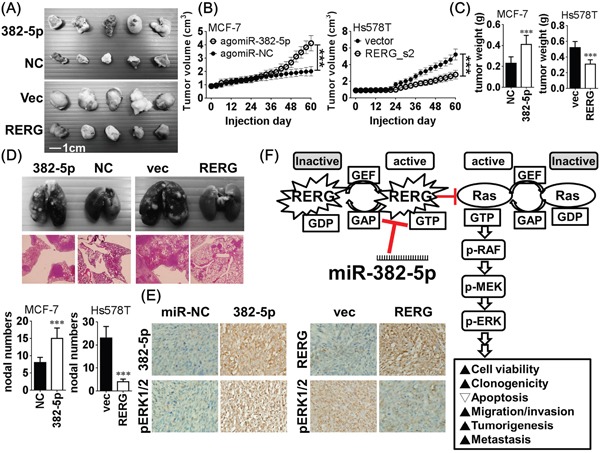
**A**. After MCF-7 cells transfected with agomiR-382-5p (5 μM) or agomiR-NC (5 μM) were injected subcutaneously and orthotopically into the axillary fossae of each female Balb/c nude mouse for 60 days (n = 5 for each group). AgomiR-382-5p significantly increased **B**. tumor volume and **C**. tumor weight compared to the agomiR-NC transfected group. In contrast, pcDNA3-RERG stably transfected Hs578T clone 2 (RERG_c2) and empty pcDNA3.1 vector transfected Hs578T cells were used for the same orthotopical inoculation, and RERG significantly reduced tumor volume and tumor weight than the vector transfected group. Data were mean ± SD of quintuple experiments (** *p* < 0.01, *** *p* < 0.001, Student *t*-test). **D**. In metastasis assays, MCF-7 cells transfected with agomiR-382-5p (5 μM) or agomiR-NC (5 μM) were injected into the lateral tail veins of each mouse for 60 days (n = 5 for each group). AgomiR-382-5p significantly increased the number of metastatic nodes in the lung compared to the agomiR-NC group. Conversely, Hs578T cells stably transfected with pcDNA3-RERG reduced the number of metastatic nodes in the lung compared to empty vector-transfected controls. Data were mean ± SD of quintuple experiments (** *p* < 0.01, *** *p* < 0.001, Student *t*-test). **E**. Representative results of *in situ* hybridization of miR-382-5p and immunohistochemistry of RERG and pERK1/2 in xenograft tumors. **F**. Schematic diagram of miR-382-5p/RERG/Ras/ERK axis in breast cancer. MiR-382-5p exerts its oncogenic role by directly targeting and repressing RERG and thereby activates the oncogenic Ras/ERK pathway to promote cell viability, clonogenicity, survival, migration, invasion and *in vivo* tumorigenesis/metastasis of breast cancer.

We evaluated the *in vivo* effects of miR-382-5p in a xenograft mouse model. We subcutaneously injected MCF-7 cells transfected with agomiR-382-5p or agomiR-NC were into the axillary fossae of female Balb/c nude mice to imitate the orthotopical circumstance. AgomiR-382-5p significantly induced higher tumor volume and tumor weight than the agomiR-NC transfected group. In contrast, Hs578T cells stably transfected with pcDNA3-RERG significantly decreased *in vivo* tumorigenicity compared to empty vector-transfected controls (Figure 6A–6C). In metastasis assays, we injected MCF-7 cells transfected with agomiR-382-5p or agomiR-NC into the lateral tail veins of each mouse (n = 5 for each group). AgomiR-382-5p significantly increased the number of metastatic nodes in the lung compared to the agomiR-NC group. Conversely, Hs578T cells stably transfected with pcDNA3-RERG reduced the number of metastatic nodes in the lung compared to empty vector-transfected controls (Figure 5D). *In situ* hybridization of miR-382-5p and immunohistochemistry of RERG and pERK1/2 in xenograft tumors revealed that miR-382-5p negatively correlated with RERG expression and positively correlated with pERK1/2 expression (Figure 5E). These data suggest that miR-382-5p promotes the *in vivo* tumorigenic and metastatic abilities of breast cancer.

## DISCUSSION

To our knowledge, this is the first study to address the regulatory role of miR-382 in breast cancer. MiR-382-5p, the primary microRNA species of miR-382, had an oncomiR role in breast cancer initiation and progression by directly targeting and repressing RERG, an estrogen-regulated RAS superfamily GTPase member, which had previously been implicated in regulating Ras/ERK signaling activation [21]. MiR-382-5p activated the oncogenic Ras/ERK pathway and promoted cell viability, clonogenicity, survival, migration, invasion and *in vivo* tumorigenesis/metastasis of breast cancer (Figure 6F). We examined the clinicopathological role for miR-382-5p in benign and breast cancer specimens and found that miR-382-5p had a diagnostic and prognostic value for its significant expression during breast cancer initiation and progression. MiR-382-5p expression negatively correlated with RERG expression, and higher miR-382-5p expression was an independent oncomiR for the higher morbidity and mortality of breast cancer. An oncogenic role for miR-382-5p has been previously reported in gastric cancer through targeting of PTEN [22]; however, contrary progression-suppressive roles of miR-382-5p have been reported in different cancer types by targeting genes of distinct pathways [23–26]. Our findings highlighted the miR-382-5p/RERG/Ras/ERK axis in breast cancer and may partially explain the different roles for miR-382-5p among different cancer types. Because miR-382-5p targeted and repressed RERG, an estrogen-regulated tumor suppressor gene, it made a connection between the oncogenic role of miR-382-5p and the estrogen-associated nature of breast cancer [27, 28].

RERG is a Ras-related small GTPase and a candidate tumor suppressor [21, 29, 30]. RERG gene expression is stimulated by both estrogen receptor (ER) α [29] and ERβ1 [31, 32], and it has been reported as a marker for ERα-positive luminal-like breast cancer and is associated with better clinical outcomes [33] or a favorable response to adjuvant tamoxifen treatment [34]. Therefore, it may provide an explanation why ERα-negative breast cancers are more invasive and metastatic than those ERα-positive counterparts [35, 36]. Loss of RERG has been implicated in the induction of breast cancer growth, but the detailed mechanism is still unclear [30]. In this study, we first reported the epigenetic regulation of the RERG gene and demonstrated that RERG overexpression resulted in deactivation of the Ras/ERK signaling effectors pRaf1, pMEK1/2, and pERK1/2. These data suggest that the tumor suppressive role of RERG was due to its negative regulation of the Ras/ERK pathway. However, we also found that Ras protein expression slightly decreased upon RERG overexpression, which complicates the potential mechanism by which RERG deactivates the Ras/ERK pathway, including through a direct Ras/RERG interaction, substrate competition, or Ras protein destabilization. Elucidation of the mechanism requires further investigation. Although ERα had been reported to activate RERG gene expression, we found that lower RERG was significantly associated with poorer OS and DFS in both ERα (+) and ERα (−) breast cancer subtypes (Supplementary Table S2 and S3). In addition, RERG has been reported to be a direct transcriptional target of ERβ1 [32], a potential tumor suppressive estrogen receptor in breast and several other cancer types [37–39], which may strengthen the estrogen inductive tumor suppressive role of RERG in breast cancer. Moreover, miR-382-5p has been reported to be a direct transcriptional target of HIF-1α [22] and TGFβ1 [40]. HIF-1α and ERβ have been reported to mutually reduce the expression and function of the other [41–43], and TGFβ1 signaling has also been reported to attenuate ERβ expression and its migration-suppressive ability [41, 44]. Previous studies suggested that HIF-1α- or TGFβ1-induced miR-382-5p expression led to the inhibition of the ERβ-RERG tumor suppressive signaling cascade in breast cancer.

In conclusion, miR-382-5p plays an oncogenic role in breast cancer initiation and progression by directly targeting and repressing RERG, thereby activating the Ras/ERK pathway to promote cancer cell viability, clonogenicity, survival, migration, invasion and *in vivo* tumorigenesis/metastasis. In addition, miR-382-5p may function as an independent diagnostic or prognostic oncomiR of breast cancer. Hence, our findings highlight the importance of the miR-382-5p/RERG/Ras/ERK axis in driving breast carcinogenesis and progression. We further propose that miR-382-5p inhibition or RERG overexpression could effectively treat breast cancers with a hyperactive Ras/ERK pathway.

## MATERIALS AND METHODS

### Subjects and tissue samples

The study population consisted of 300 breast cancer cases and 300 benign breast disease controls who were treated at Tri-Service General Hospital (TSGH) between 1992.06 and 2007.12. The recruited benign breast diseases comprised 237 nonproliferative lesions (NP, 124 fibroadenoma, 107 fibrocystic changes, 6 adenosis) and 63 proliferative diseases without atypia (PDWA, 44 mild ductal hyperplasia and 19 sclerosing adenosis), which have a lower relative risk of breast cancer [46]. The breast cancer cases were comprised of 61 ductal carcinoma *in situ*, 27 infiltrating ductal carcinoma (IDC) grade I (GI), 110 IDC GII, 74 IDC GIII, 18 infiltrating lobular carcinoma (ILC), 7 colloid carcinoma, and 3 medullary carcinoma. In accordance with the TSGH Institutional Review Board guidelines (TSGH-IRB-093-05-00004, 097-05-008, 098-05-204, 098-05-311, 099-05-273, and 100-05-236), patients were followed for at least eight years postoperative follow-up. Clinical information was obtained from patient charts and pathological reports. Tumor staging and pathological grading were recorded according to the American Joint Committee on Cancer (AJCC) 7th tumor-node-metastasis (TNM) staging system and the Modified Bloom-Richardson histologic grading criteria, respectively.

### MiRNA microarray and bioinformatics prediction

For miRNA microarray assays, 5 fresh breast cancer sample specimens (one infiltrating ductal carcinoma (IDC) grade I, two IDC grade II and two IDC grade III (tow)) and 5 normal breast samples from benign breast diseases (two fibrocystic changes and three fibroadenomas) were microdissected for RNA extraction. MiRNA microarray was performed with Agilent Human 8×15K miRNA Microarrays Rel18.0, and the microarray data were analyzed using an Agilent Certified Service Provider Program (Welgene Biotech Co., Ltd., Taiwan). Among the upregulated miRNAs, miR-382-5p revealed the highest tumor/normal ratio (Supplementary Table S1). Using bioinformatic target gene prediction with miRBase and TargetScanHuman, 27 candidate target genes were predicted in both databases, but only *RERG* had been reported as a putative tumor suppressor gene (Supplementary Figure 1A).

### Cell lines

The MCF-7, T-47D, BT-474, MDA-MB-231, Hs578Th human breast cancer cell lines and H184B5F5/M10 nontumorigenic human breast epithelial cell line were originally obtained from the Bioresource Collection and Research Center. MDA-MB-231 and Hs578T cells were maintained in DMEM containing 10% fetal bovine serum, 1 μg/ml penicillin and 1 μg/ml streptomycin (Invitrogen) at 37°C in a 5% CO_2_ atmosphere. T-47D and BT-474 cells were maintained in RPMI-1640, and MCF-7 and H184B5F5/M10 cells were maintained in MEM-α with the same supplements and culture conditions. For stable clone selection of pcDNA3-RERG-transfected cells, 400 μg/mL G418 (A1720, Sigma-Aldrich Co.) was used for 30-day selection and 200 μg/mL G418 was used for maintenance.

### RNA preparation and quantitative real-time PCR

Total RNA of treated cells was isolated by TRIzol reagent (Invitrogen) and treated with RQ1 RNase-free DNase (Promega) according to the manufacturer's instructions to remove genomic contamination. For stem-loop based microRNA real-time PCR, five micrograms of treated RNA was subjected to reverse transcription with SuperScript III (Invitrogen). SYBR Green-based quantitative real-time PCR was performed by StepOne Real-Time PCR System (Applied Biosystems) using 2× hot start PCR master mix (Applied Biosystems), and U6 was used as an internal control. Each condition was run in six replicates.

### Western blot analysis

After washing with phosphate-buffered saline (PBS), treated cells were lysed in 200 μl RIPA lysis buffer (Millipore) containing protease inhibitor (Roche). A total of 30 μg protein from cell lysates was loaded onto an SDS polyacrylamide gel followed by western blot analysis to compare the protein level of the indicated genes (RERG: Proteintech 10687-1, Ras: Cell Signal Tech.#3339, Raf1: Cell Signal Tech.#9422, pRaf1 (S338): Cell Signal Tech.#9427, MEK1: Cell Signal Tech.#12671, pMEK1 (S218/222): Abcam ab32088, ERK1: Abcam ab9363, ERK2: Abcam ab32081, pERK1/2 (T202/Y204): Cell Signal Tech.#9422, GAPDH: Epitomics #2251). The immuno-reactive bands were revealed by ECL (Millipore) then developed using a UVP BioSpectrum Imaging System, and each condition was performed in triplicate.

### *In situ* hybridization

Slides were deparaffinized and treated with 3% H_2_O_2_ for 10 min at room temperature to block endogenous peroxidase. Slides were retrieved with proteinase XIV (0.125 ng/ml) for 1 h at 37°C following fixation in 3.7% formaldehyde for 10 min at room temperature. Prepared slides were transferred to a standard prehybridization solution (50% deionized formamide, 12.5% dextran sulfate, 0.3 M NaCl, 10 mM Tris-HCl pH 6.5, 5 mM EDTA, 0.1 M NaH_2_PO_4_, 1 mg/ml tRNA (Invitrogen), 1× Denhardt's solution (Sigma) in 1% DEPC treated H_2_O) for 1 h at 80°C to eliminate RNase activity. Pre-hybridizations were performed at 55°C for 1 h in a shaking hybridization oven. A total of 100 pmol of probe (5′-Biotin-CGAATCCACCACGAACAACTTC) was denatured at 99°C for 10 min followed by hybridization at 55°C overnight. After washing three times in 0.2 × SSC at 55°C each for 1 h and rinsing with DEPC-treated PBS for 10 min, each slide was incubated with HRP-conjugated Avidin (Dako) at room temperature for 1 h, incubated with DAB chromogen (Thermo Scientific) for 10 min and counterstained with hematoxylin.

### Immunohistochemistry

Immunohistochemistry was performed in a constructed TMA containing 100 individual 2 mm-diameter specimens per array. Each slide of 4 μm dissected TMA was blocked with 10% goat serum for 1 h and incubated with the indicated primary antibodies (RERG: Proteintech 10687-1, pERK1/2 (T202/Y204): Cell Signal Tech.#9422) for 2 h at room temperature. After washing three times in TBST (10 mM Tris pH 7.4, 150 mM NaCl, 0.1% Tween-20) for 10 min, slides were processed according to the Super Sensitive Polymer HRP Detection System/DAB kit instructions (Thermo Scientific) and counterstained with hematoxylin.

### MTT assay

Cells were transfected with the indicated small RNA (miR-382-5p mimics, miR-382-5p inhibitors, miRNA mimics negative control (mNC), miRNA inhibitors negative control (iNC), RERG siRNAs, scrambled control siRNA (SC), Ribobio Co, Guangzhou, China) for 72 h. After washing twice in PBS, 3000 cells from each treatment were seeded in a 96-well plate overnight, and an MTT assay was performed to detect cell viability. In brief, 20 μl of 5 mg/ml MTT reagent was added to each well and incubated at 37°C for 3.5 h before reading the absorbance at 570 nm (A570). A570 was recorded at 0 h, 24 h, 48 h and 72 h, and each condition was performed with six replicates.

### Flow cytometry

After trypsinizing and washing, 1 × 10^6^ cells were fixed in 100% ethanol for 10 min and incubated with 1 mg/ml propidium iodide (Sigma-Aldrich) for 10 min at room temperature. Cells were analyzed within 20 min of staining on a BD FACSCalibur (BD Biosciences), and six repeats were performed for each condition.

### Colony-formation assay

MiR-382-5p mimics versus mNC and miR-382-5p inhibitors versus iNC treated cells were used in colony-formation assay. In brief, 0.5 ml of 0.5% agarose in complete medium was used as the bottom agar in a 24-well plate, and 1 × 10^4^ cells were mixed with 0.3% agarose in complete medium 48 h after transfection. Cells were maintained in a humidified 5% CO_2_ incubator at 37°C for 15 days with fresh medium replacement every three days. Cells were stained with crystal violet for 1 min and destained with tap water for 15 min. Colonies were counted using Image J software for each well, and triplicate repeats were performed for each condition.

### Migration/invasion assays

Transwell migration assays were performed on 8 μm inserts (BD Biosciences) with 1 × 10^4^ cells from each condition. Transwell invasion assays were performed using the same inserts coated with 1 mg/ml Matrigel (Invitrogen) containing 2 × 10^4^ cells for each condition. Migration and invasion chambers were incubated in a humidified 5% CO_2_ incubator at 37°C for 48 h. Cells were fixed with methanol, and the inner surface of the upper chambers was wiped with cotton swabs to remove cells that had not migrated. After washing, the chambers were stained with crystal violet, and the transwell membranes were removed and mounted on slides. The crystal violet-stained area was analyzed using Image J software, and five random fields were counted at 100 × magnification. Each condition was performed with in six replicates.

### Plasmid construction and luciferase reporter assay

The human RERG open-reading frame was amplified by PCR from human genomic DNA using the following forward primer with a *Hind*III restriction site: 5′- cctcctaagcttGTCTACCAACACCCATCATG -3′ and the following reverse primer with a *Xho*I restriction site: 5′- cctcctgagctcAGCTGGGCTGCCTA -3′. The 652 bp PCR product was digested with *Hin*dIII/*Xho*I and inserted into pcDNA3.1/His A vector (Invitrogen). The RERG 3′UTR containing the miR-382-5p binding site was amplified by PCR from human genomic DNA using the following forward primer containing a *Spe*I restriction site: 5′- cctcctactagtGATGCTCACCAAAATCAGTAGTTAG -3′, and the following reverse primer containing a *Hin*dIII restriction site: 5′- ATGACAGAATGAGAAAATTATGCTTaagcttcctcc -3′. The 1353 bp PCR product was digested with *Spe*I/*Hin*dIII and inserted into pMIR-Reporter vector (Invitrogen). All constructs were verified by auto-sequencing.

For luciferase reporter assays, a total of 2 × 10^5^ MCF-7 or Hs578T cells were seeded into 6 cm dishes 16 h before transfection. A total of 120 pmol miR-382-5p mimics or miRNA mimic negative controls were co-transfected with 1 μg pMIR-Reporter empty vector, pMIR-Reporter carrying the wild-type RERG-3′UTR, or pMIR-Reporter carrying a mutant RERG-3′UTR using Lipofectamine 2000 (Invitrogen). For each combination, 500 ng of β-gal Control vector was also co-transfected as an internal control. Luciferase assays were performed with a Dual-Light Luciferase & β-Galactosidase Reporter Gene Assay System (Invitrogen) according to the manufacturer's instructions, and each condition was performed with six replicates.

### *In vivo* tumor xenograft and metastasis assays

Female athymic Balb/c nude mice (6-weeks old) were purchased from the National Laboratory Animal Center and allowed to acclimate for 1 week under conditions approved by the Laboratory Animal Center of National Defense Medical Center. The *in vivo* tumorigenic effects of miR-382-5p were evaluated in a xenograft model. In brief, 1 × 10^7^ MCF-7 cells transfected with agomiR-382-5p (5 μM) or agomiR-NC (5 μM, Ribobio Co) were suspended in 100 μl PBS for each mouse and subcutaneously and orthotopically injected into the axillary fossae of the female nude mice (5 mice per group). Tumor diameters were measured every 3 days. Mice were sacrificed 60 days after injection, and tumors were weighed after necropsy. Tumor volume was calculated as follows: length × width^2^ × 1/2.

The protocol of this study is according to the Training Guildline for Researchers to Hold Animals of the National Defense Medical Center Laboratory Animal Center. And the statement of the euthanization of tumor-inoculated animals was described as followed (in italics).

*Animal inoculated with tumor cells should be euthanized if any of the following conditions is observed*.

1. *Tumor ulceration occurs on animal*.
2. *Animal is unable to present normal activities due to the tumor*.
3. *Tumor weight exceed 10% of the animal weight*.
4. *Animal abdomen appears dark-gray/green or ascites exceed 20% of the animal weight*.
5. *Lethargy, anorexia, dehydration, or other sign of obvious stress or pain*.
6. *Animal is unable to feed or drink normally due to the tumor*.

*If rupture or ulceration of tumor occurs before the expected size is achieved, experiment design or strategy should be reviewed instead of leaving it for continual tumor growth*.

*Animal inoculated with tumor cells are suggested to be euthanized when the average diameter of tumor was larger than 20 mm in a 25-g mouse or 40 mm in a 250-g rat*.

In current work (according to the protocol), each average diameter of 20 mm was not surpassed. In our future work, we will limit tumor size to 2 cm^3^ in any future study of tumor inoculation.

For *in vivo* pulmonary metastasis assays, 2 × 10^7^ MCF-7 cells were transfected with agomir-382-5p (5 μM) or agomiR-NC (5 μM). The cells were injected into the lateral tail veins of nude mouse (5 mice per group). Mice were sacrificed 60 days after injection, and xenograft tumors and lungs were fixed in phosphate-buffered neutral formalin before paraffin embedding. Sections 4-μm thick were stained with hematoxylin and eosin or used for immunohistochemistry. The same cell numbers and strategies were used to evaluate *in vivo* tumor xenograft and metastastic effects of RERG, pcDNA3-RERG stably transfected Hs578T clone 2 (RERG_c2) and empty pcDNA3.1 vector transfected Hs578T cells were used for orthotopical inoculation or injection into the lateral tail veins of nude mice as described above. The number of metastatic nodes in the lung of each mouse was counted from ten serial H&E-stained slides of both right and left lung lobes under low power (magnification × 40) fields to count the mean number of nodes on each side.

### Data analysis

Original real-time PCR data were quantified with StepOne Software Ver.2.2.2, and western blot data were quantified with Image J software. The data were recorded as continuous variants and analyzed with Student's *t* test. *In situ* hybridization and immunohistochemistry data were quantified with Aperio ImageScope and Spectrum Ver. 10.0. Univariant and multivariant analyses of miR-382-5p, RERG and breast cancer risk factors were evaluated using logistic regression. MiR-382-5p and RERG expression data were categorized by the mean value, indicated in Table 1 and Table 2. Survival assays were evaluated using a Kaplan-Meier curve with log-rank test and Cox proportional hazard regression model in breast cancer patients. Statistical analyses were performed using SPSS 16.0 and Excel 2010. All statistical tests and *p* values were two-sided, and the level of significance was set to < 0.05 (*), < 0.01 (**), or < 0.001 (***).

## SUPPLEMENTARY TABLES AND FIGURE




