# Supplementary material for: MicroRNA-382-5p aggravates breast cancer progression by regulating the RERG/Ras/ERK signaling axis
Source: Oncotarget. 2016 Sep 29;8(14):22443–59. doi: 10.18632/oncotarget.12338 (PMC5410235; doi:10.18632/oncotarget.12338)
Supplement: Supplementary file 1 [file oncotarget-08-22443-s001.pdf]

MicroRNA-382-5p aggravates breast cancer progression by regulating the RERG/Ras/ERK signaling axis

SUPPLEMENTARY TABLES AND FIGURES

Supplementary Table S1: Top 15 distinct miRNAs between breast cancer and normal breast tissue (microarray data)

	Breast cancer	Normal breast	Cancer/normal	*p*
Onco-miR				
hsa-miR-382-5p	254.91	0.11	2317.36	<0.001
hsa-miR-410	100.57	0.12	838.08	<0.001
hsa-miR-1254	64.47	0.15	429.80	<0.001
hsa-miR-381	137.43	0.71	192.48	<0.001
hsa-miR-154	95.42	0.58	165.95	<0.001
hsa-miR-136-3p	75.54	1.48	50.90	<0.001
hsa-miR-654-3p	163.12	11.44	14.26	<0.001
hsa-miR-92a-1	512.79	39.67	12.93	<0.001
hsa-miR-21-3p	68.20	5.35	12.75	<0.001
hsa-miR-204	66.05	5.57	11.86	<0.001
hsa-miR-155	81.66	7.43	10.99	<0.001
hsa-miR-21-5p	31540.60	3171.58	9.94	<0.001
hsa-miR-125b-2	138.81	16.32	8.51	<0.001
hsa-miR-542-3p	97.29	12.89	7.55	<0.001
hsa-miR-214	74.95	12.13	6.18	<0.001
Tumor suppressive miRNA				
hsa-miR-550a-3p	0.08	16.44	0.0049	<0.001
hsa-miR-15b	0.07	12.59	0.0056	<0.001
hsa-miR-132	0.09	10.48	0.0086	<0.001
hsa-miR-212	0.08	8.44	0.0095	<0.001
hsa-miR-153	0.08	8.13	0.0098	<0.001
hsa-miR-200c	0.09	7.21	0.0125	<0.001
hsa-miR-924	0.11	7.61	0.0145	<0.001
hsa-miR-105	0.12	6.28	0.0191	<0.001
hsa-miR-190b	0.11	5.30	0.0208	<0.001
hsa-miR-552	0.11	5.07	0.0217	<0.001
hsa-miR-145	0.28	5.47	0.0512	<0.001
hsa-miR-7-1	0.37	4.80	0.0771	<0.001
hsa-miR-143	0.44	5.12	0.0859	<0.001
hsa-miR-1301	0.95	4.43	0.2144	<0.001
hsa-miR-1244	1.10	4.60	0.2391	<0.001

Supplementary Table S2: Survival assay of miR-382-5p, RERG and clinical prognostic factors of ER (+) breast cancer

See Supplementary File 1

Supplementary Table S3: Survival assay of miR-382-5p, RERG and clinical prognostic factors of ER (-) breast cancer

See Supplementary File 1

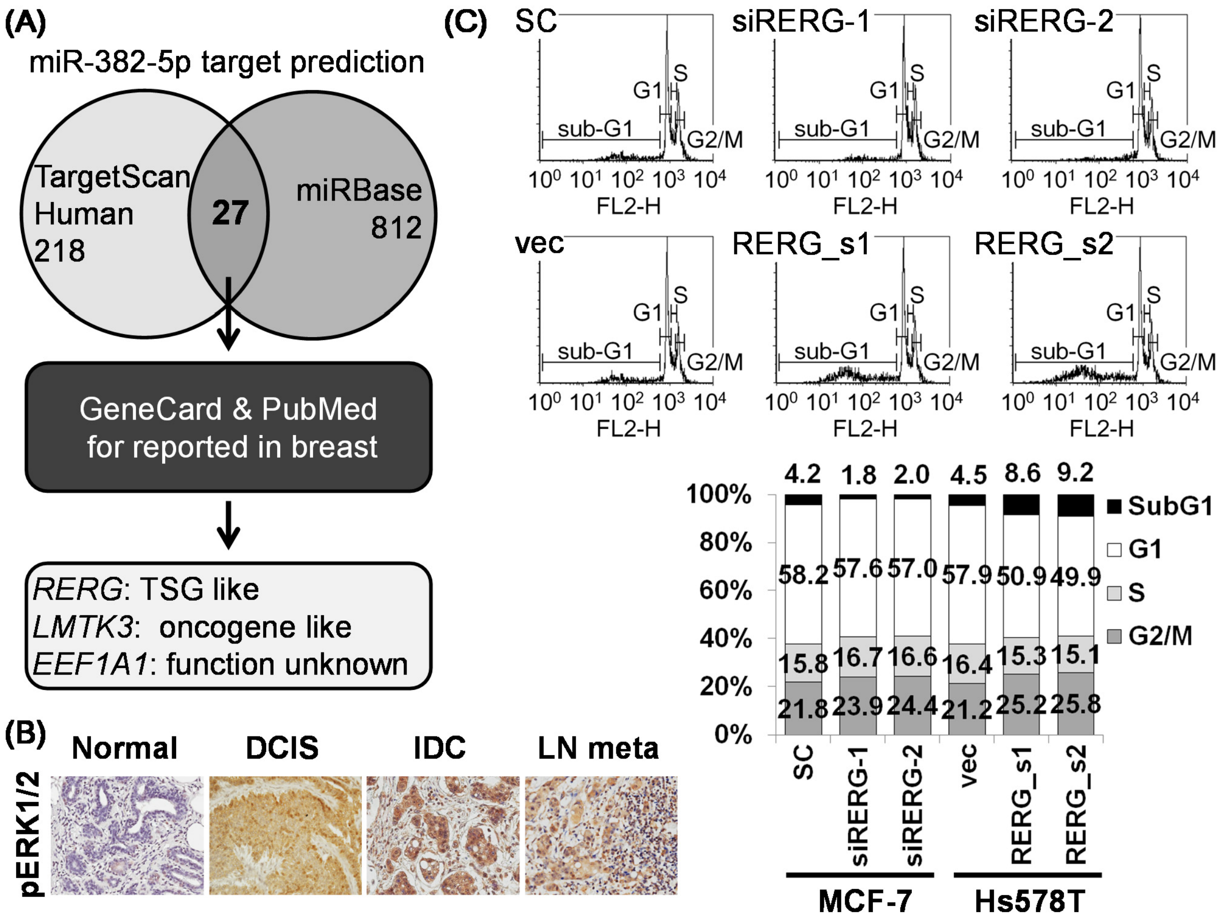

Supplementary Figure S1: A. Potential target genes of miR-382-5p were predicted with TargetScanHuman and miRBase, and there were 27 candidate target genes predicted in both databases. After searching each gene in GeneCard and PubMed, three genes had been reported in breast tissues, i.e. *RERG*, *LMTK3* and *EEF1A1*, and only *RERG* had been reported as a potential tumor suppressor gene. **B.** The levels of pERK1/2 were detected by immunohistochemistry in formalin-fixed paraffin-embedded breast specimens. Representative pERK1/2 expression patterns were showed among of benign breast disease, carcinoma *in situ*, primary breast cancer and lymph node metastatic breast cancer. **C.** MCF-7 cells were transfected with 120 pmol scrambled miR control (SC), 120 pmol siRERG-1, and 120 pmol siRERG-2 for 72 h, and the sub-G1 cell population of PI-stained flow cytometry was counted. Those results indicated that RERG knockdown reduced sub-G1 cell population. Conversely, two pcDNA3-RERG stably transfected Hs578T clones (RERG_s1 and RERG_s2) and empty vector transfected control (vec) also analyzed with PI-stained flow cytometry, and RERG overexpression increased sub-G1 cell population.
