# Supplementary material for: MicroRNA-382-5p aggravates breast cancer progression by regulating the RERG/Ras/ERK signaling axis
Source: Oncotarget. 2016 Sep 29;8(14):22443–59. doi: 10.18632/oncotarget.12338 (PMC5410235; doi:10.18632/oncotarget.12338)
Supplement: Supplementary file 2 [file oncotarget-08-22443-s002.docx]

**Supplementary Table S2. Survival assay of miR-382-5p, RERG and clinical prognostic factors of ER (+) breast cancer**

|  |  | Overall survival | | | | Disease-free survival | | | |
| --- | --- | --- | --- | --- | --- | --- | --- | --- | --- |
|  |  | Univariate ^a^ | | Multivariate ^a^ | | Univariate ^a^ | | Multivariate ^a^ | |
|  | n | HR (95% CI) *^a^* | *p* | HR (95% CI)*^a^* | *p* | HR (95% CI) *^a^* | *p* | HR (95% CI)*^a^* | *p* |
| miR-382-5p low | 100 | 1 (ref) |  | 1 (ref) |  | 1 (ref) |  | 1 (ref) |  |
| high | 107 | **2.462 (1.348-4.497)** | **0.003** | **1.936 (1.103-4.431)** | **0.041** | **2.217 (1.387-3.546)** | **0.001** | **2.327 (1.213-4.462)** | **0.011** |
| RERG high | 113 | 1 (ref) |  | 1 (ref) |  | 1 (ref) |  | 1 (ref) |  |
| low | 94 | **1.749 (1.006-3.055)** | **0.048** | 1.127 (0.534-2.377) | 0.754 | **1.549 (1.010-2.415)** | **0.047** | 1.530 (0.822-2.846) | 0.179 |
| Age ≦53 y/o | 123 | 1 (ref) |  |  |  | 1 (ref) |  |  |  |
| ＞53 y/o | 84 | 0.850 (0.486-1.486) | 0.569 | - | - | 0.933 (0.595-1.462) | 0.761 | - | - |
| Family history (-) | 119 | 1 (ref) |  |  |  | 1 (ref) |  |  |  |
| (+) | 20 | 1.007 (0.350-2.894) | 0.637 | - | - | 1.044 (0.441-2.475) | 0.921 | - | - |
| unknown | 69 | **-** | **-** | **-** | **-** | **-** | **-** | **-** | **-** |
| TNM stage 0+I+II | 167 | 1 (ref) |  | 1 (ref) |  | 1 (ref) |  | 1 (ref) |  |
| III+IV | 40 | **11.563 (6.513-20.529)** | **<0.001** | **8.270 (4.481-15.260)** | **<0.001** | **7.449 (4.688-11.834)** | **<0.001** | **5.831 (3.530-9.630)** | **<0.001** |
| Histologic grade CIS+G1 | 65 | 1 (ref) |  | 1 (ref) |  | 1 (ref) |  | 1 (ref) |  |
| G2+G3 | 142 | **6.269 (2.258-17.408)** | **<0.001** | 2.555 (0.856-7.624) | 0.093 | **3.590 (1.897-6.794)** | **<0.001** | 1.860 (0.934-3.703) | 0.077 |
| PR (+) | 172 | 1 (ref) |  |  |  | 1 (ref) |  |  |  |
| (-) | 35 | 1.671 (0.875-3.193) | 0.120 | - | - | 1.718 (0.859-2.880) | 0.184 | - | - |
| HER2 (+) | 66 | 1 (ref) |  |  |  | 1 (ref) |  |  |  |
| (-) | 141 | 0.763 (0.433-1.346) | 0.351 | - | - | 0.721 (0.457-1.136) | 0.158 | - | - |
| Chemotherapy (-) | 97 | 1 (ref) |  |  |  | 1 (ref) |  |  |  |
| (+) | 110 | 0.707 (0.400-1.247) | 0.231 | - | - | 0.708 (0.449-1.115) | 0.136 | - | - |
| Radiotherapy (-) | 118 | 1 (ref) |  |  |  | 1 (ref) |  |  |  |
| (+) | 89 | 0.882 (0.505-1.541) | 0.659 | - | - | 1.113 (0.715-1.734) | 0.635 | - | - |
| Hormone therapy (-) | 63 | 1 (ref) |  |  |  | 1 (ref) |  |  |  |
| (+) | 114 | 1.255 (0.701-2.247) | 0.443 | - | - | 1.163 (0.723-1.869) | 0.533 | - | - |

^a^ Based on Cox regression model, statistical significance (*p* < 0.05) is shown in bold.

TNM, tumor node metastasis; HR, hazard ratio; CI, confidence interval.

^b^ Categorization was also stratified as low (≤mean) and high (>mean). Abbrev: HR: hazard ratio, CI: confidence interval.

**Supplementary Table S3. Survival assay of miR-382-5p, RERG and clinical prognostic factors of ER (-) breast cancer**

|  |  | Overall survival | | | | Disease-free survival | | | |
| --- | --- | --- | --- | --- | --- | --- | --- | --- | --- |
|  |  | Univariate ^a^ | | Multivariate ^a^ | | Univariate ^a^ | | Multivariate ^a^ | |
|  | n | HR (95% CI) *^a^* | *p* | HR (95% CI)*^a^* | *p* | HR (95% CI) *^a^* | *p* | HR (95% CI)*^a^* | *p* |
| miR-382-5p low | 32 | 1 (ref) |  | 1 (ref) |  | 1 (ref) |  | 1 (ref) |  |
| high | 61 | **4.403 (1.281-15.126)** | **0.019** | **2.723 (1.015-12.575)** | **0.048** | **3.662 (1.316-12.618)** | **0.004** | **2.816 (1.021-11.515)** | **0.047** |
| RERG high | 34 | 1 (ref) |  | 1 (ref) |  | 1 (ref) |  | 1 (ref) |  |
| low | 59 | **3.131 (1.043-9.400)** | **0.042** | 1.593 (0.408-6.216) | 0.503 | **3.973 (1.153-13.697)** | **0.029** | 1.932 (0.475-7.862) | 0.358 |
| Age ≦53 y/o | 51 | 1 (ref) |  |  |  | 1 (ref) |  |  |  |
| ＞53 y/o | 42 | 2.489 (0.913-6.111) | 0.074 | - | - | 1.043 (0.419-2.600) | 0.928 | - | - |
| Family history (-) | 55 | 1 (ref) |  |  |  | 1 (ref) |  |  |  |
| (+) | 9 | 2.248 (0.296-17.069) | 0.434 | - | - | 1.531 (0.195-11.995) | 0.685 | - | - |
| unknown | 30 | **-** | **-** | **-** | **-** | **-** | **-** | **-** | **-** |
| TNM stage 0+I+II | 81 | 1 (ref) |  | 1 (ref) |  | 1 (ref) |  | 1 (ref) |  |
| III+IV | 12 | **5.157 (1.949-13.641)** | **0.001** | **4.397 (1.654-11.692)** | **0.003** | **4.886 (1.843-12.951)** | **0.001** | **5.081 (1.913-13.492)** | **0.001** |
| Histologic grade CIS+G1 | 27 | 1 (ref) |  |  |  | 1 (ref) |  |  |  |
| G2+G3 | 66 | 1.057 (0.404-2.763) | 0.910 | - | - | 2.258 (0.658-7.750) | 0.196 | - | - |
| PR (+) | 60 | 1 (ref) |  |  |  | 1 (ref) |  |  |  |
| (-) | 33 | 2.716 (0.912-6.585) | 0.072 | - | - | 1.119 (0.440-2.844) | 0.813 | - | - |
| HER2 (+) | 63 | 1 (ref) |  |  |  | 1 (ref) |  |  |  |
| (-) | 30 | 0.705 (0.256-1.944) | 0.501 | - | - | 0.820 (0.323-2.085) | 0.677 | - | - |
| Chemotherapy (-) | 40 | 1 (ref) |  |  |  | 1 (ref) |  |  |  |
| (+) | 53 | 2.201 (0.897-5.398) | 0.085 | - | - | 1.733 (0.704-4.266) | 0.232 | - | - |
| Radiotherapy (-) | 53 | 1 (ref) |  |  |  | 1 (ref) |  |  |  |
| (+) | 40 | 0.727 (0.296-1.784) | 0.487 | - | - | 0.609 (0.244-1.515) | 0.286 | - | - |
| Hormone therapy (-) | 57 | 1 (ref) |  |  |  | 1 (ref) |  |  |  |
| (+) | 36 | 1.639 (0.682-3.941) | 0.269 | - | - | 1.149 (0.462-2.859) | 0.765 | - | - |

^a^ Based on Cox regression model, statistical significance (*p* < 0.05) is shown in bold.

TNM, tumor node metastasis; HR, hazard ratio; CI, confidence interval.

^b^ Categorization was also stratified as low (≤mean) and high (>mean). Abbrev: HR: hazard ratio, CI: confidence interval.
